# The Zebrafish Model in Animal and Human Health Research

**DOI:** 10.3390/ijms26051945

**Published:** 2025-02-24

**Authors:** Marta Anna Szychlinska, Antonella Marino Gammazza

**Affiliations:** 1 Department of Precision Medicine in Medical, Surgical and Critical Care (MEPRECC), University of Palermo, 90127 Palermo, Italy; 2 Department of Biological, Chemical and Pharmaceutical Sciences and Technologies (STEBICEF), University of Palermo, 90127 Palermo, Italy; antonella.marinogammazza@unipa.it

The present Special Issue aims to highlight several advantages of the zebrafish model in various fields of biomedical and ecotoxicological research. Its contributions report the latest discoveries, methods, and technological advancements related to this research, enabling us to further understand the molecular aspects of several pathologies such as developmental disorders, mental disorders, and metabolic diseases. Moreover, it supports the usefulness of the model in environmental toxicant assessments, the discovery of novel therapeutic approaches, and drug screening.

Zebrafish (Danio rerio) have become a widely used vertebrate model for scientific research. In particular, they is used as a model to study vertebrate development and disease, physiology, behavior, toxicology, oncology, and drug discovery [1]. This is mainly due to the numerous advantages of the model over rodent models, including high fertility rate (hundreds of embryos in a single clutch), small size, rapid development, and optical transparency during early development, allowing for live imaging at the organism level [1]. Another important advantage is related to its strong genetic correspondence with humans, for which zebrafish become particularly useful both to model human diseases and to assess the effects of environmental contaminants on human health. In addition, the use of tissue-specific transgenic animals, simply generated under the control of various selected gene promoters, allow for the live imaging and tracking of cellular dynamics to study the molecular mechanisms underlying organ development, as well as the pathological features of the related diseases [2]. This aspect has been further highlighted in a review by Alberti et al. [3], underlying the relevant potential of transgenic and xenograft zebrafish models in glioblastoma cancer research (Figure 1).

Likewise, zebrafish possess metabolic characteristics similar to humans, becoming a fascinating animal model for understanding the human pathogenesis of metabolic diseases and identifying potential therapeutic options. In this regard—and, in particular, in diabetes research—zebrafish have demonstrated several features such as similar fat metabolism and blood glucose regulation mechanisms when compared to mammals [4,5]. This aspect has been exploited in the interesting study by Ge et al. [6], in which the induction of zebrafish diabetes was achieved by overfeeding. The obtained results permit the identification of novel molecular factors that contribute to lipid droplet accumulation and insulin resistance, further elucidating the molecular aspects of the pathology and paving the way for the discovery of novel therapeutic approaches.

**Figure 1:**
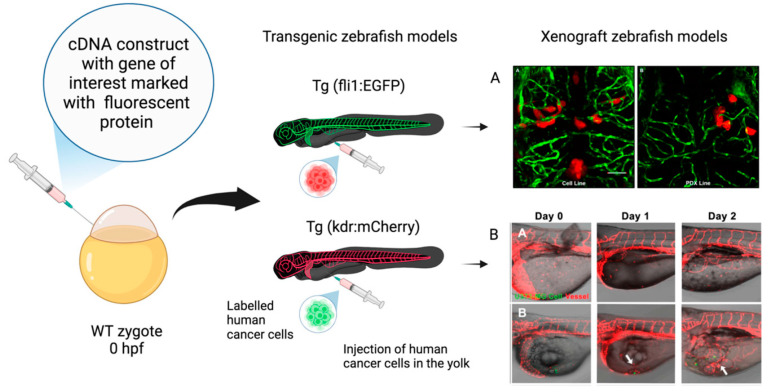
Schematic representation of transgenic zebrafish models and xenograft models used in GBM research. (**A**) Confocal images of zebrafish Tg (fli1a:EGFP)y1 larvae brains injected with various GBM cell lines. (**AA**) Adult D54-MG-tdTomato cell line (red) and (**AB**) D2159MG pediatric xenoline (red). [Adapted with permission from Umans et al., 2021 [7]]. Copyright 2021, American Chemical Society. (**B**) Confocal images of zebrafish Tg(kdr:mcherry) larvae xenotransplantation model of U373-MG tumorsphere cells (green, white arrows). [Adapted with permission from Lai et al., 2017 [8]]. Copyright: © 2017 Lai et al. The entire figure reported with permission from Alberti et al., 2024 [3]. Copyright: © 2024 Alberti et al.

Furthermore, zebrafish are a very valuable model in all areas of toxicology, including studies evaluating the biological effects of chemicals and toxic compounds in the natural environment. Zebrafish are increasingly being employed in predictive toxicology, in the screening of contaminants, and in the assessment of environmental matrices, permitting monitoring and, finally, allowing researchers to find adequate preventive strategies to preserve human and animal health [9].

In an interesting review by Stachurski et al. [10], the zebrafish model is discussed as a promising tool in ecotoxicity research dealing with toothpaste’s environmental impact. The validity of the model in this field has been further demonstrated in a study by Seli et al. [11], evaluating the influence of salts introduced into ecosystems by human activity. In particular, the study examined how changes in the salinity concentration would affect the survival and development of zebrafish from the two-cell to the blastocyst stage and from the blastocyst to the larval stage. The results demonstrate that increased NaCl concentrations alter gene expression and cause morphological abnormalities, especially those implicated in swim bladder development, suggesting salinity-related developmental toxicity (Figure 2).

Another pollutant resulting from human activity, investigated here by employing zebrafish embryos, is represented by non-combustion-derived magnetite, a portion of the coarse particulate matter, contributing to air and water pollution in urban settings. The neurotoxicity related to its exposure has been investigated in a study by Cacialli et al. [12]. The results of this study suggest that magnetite has deleterious effects on zebrafish brain development, triggered by the induction of apoptosis, oxidative stress, and inflammation, resulting in decreased neurogenesis. The study highlights the validity of the model in neurotoxicological research.

Likewise, endocrine disruptors, another class of environmental pollutants, have been recently associated with the increase risk of onset and progression of neurodevelopmental disorders such as autism spectrum disorder and schizophrenia. In this context, zebrafish have been widely used to model neurodevelopmental disorders and to analyze the related behavioral and phenotypic alterations [13]. In a study by Wang et al. [14], the zebrafish model of neurodevelopmental disorders has been established by deleting the adnp gene family using CRISPR/Cas9 technology. The mutant zebrafish demonstrated impaired neuron proliferation, differentiation, and function, leading to cerebellar underdevelopment, atrophy, and behavioral changes. These latter were demonstrated to be further aggravated by the exposure of mutant zebrafish to endocrine disruptors, suggesting the co-contribution of genetic and environmental factors in neurodevelopmental impairments (Figure 3).

In toxicological research, the zebrafish model also represents a valuable tool for drug safety testing and screening. In this context, the in vivo evaluation of the biological effects and biosafety of these products is essential, and zebrafish provides an ideal platform for high-throughput toxicological analysis, allowing us to minimize the use of mammalian models without losing reliability [15]. For instance, in the study by Reina et al. [16], the biological effects of a conditioned medium derived from Wharton’s jelly mesenchymal stem cells have been evaluated on zebrafish embryos. The results report that the exposure of zebrafish to non-lethal doses of the conditioned medium promote the upregulation of genes involved in antioxidant defense, glycolysis, and cell survival, together with the downregulation of pro-apoptotic genes, suggesting protective biological effects on zebrafish development. Moreover, the high similarity between z ebrafish and other vertebrates in terms of brain structure and function, as well as neurochemical and behavioral systems, makes this model just as interesting and valuable for studying therapeutic strategies for neurological diseases such as anxiety-related disorders [16]. In a study by Widelski et al. [17], the anxiolytic activity of three simple coumarins (officinalin, stenocarpin isobutyrate, and officinalin isobutyrate) has been tested in zebrafish larvae. The results of the study demonstrate the anxiolytic activity of coumarins through behavioral tests, as well as their influence on the expression of genes related to anxiety onset and extinction, further highlighting the utility of the zebrafish model in therapeutic drug discovery and screening.

## Figures and Tables

**Figure 2:**
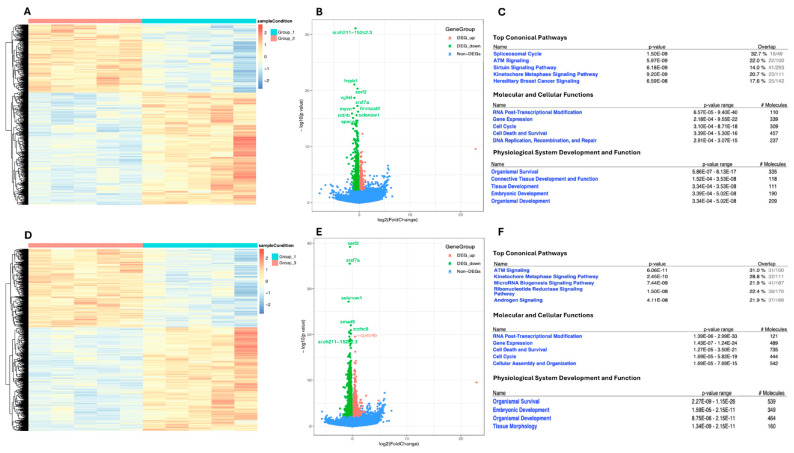
Gene expression alteration in zebrafish raised in heightened NaCl concentrations. (**A**,**D**) Heatmap (red representing high and blue representing low) illustration showing genes differentially expressed between zebrafish raised in different NaCl concentrations. (**B**,**E**) Volcano plots (red spot represents upregulation, green spot represents downregulation) for RNA seq of zebrafish comparing those raised in different concentrations of NaCl to those raised in embryo medium (control). (**C**,**F**) Table showing noteworthy genetic change from zebrafish grown in embryo medium to those grown in different concentrations of NaCl. #: number of molecules. Reported with permission from Seli et al., 2024 [11]. Copyright: © 2024 Seli et al.

**Figure 3:**
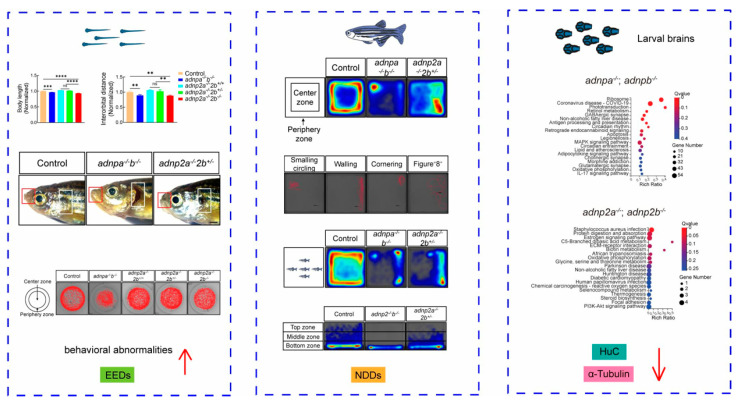
Graphical abstract reported with permission from Wang et al., 2024 [14]. Copyright: © 2024 Wang et al.
